# Lung Toxicity of Ambient Particulate Matter from Southeastern U.S. Sites with Different Contributing Sources: Relationships between Composition and Effects

**DOI:** 10.1289/ehp.9234

**Published:** 2006-06-12

**Authors:** JeanClare Seagrave, Jacob D. McDonald, Edward Bedrick, Eric S. Edgerton, Andrew P. Gigliotti, John J. Jansen, Lin Ke, Luke P. Naeher, Steven K. Seilkop, Mei Zheng, Joe L. Mauderly

**Affiliations:** 1 Lovelace Respiratory Research Institute, Albuquerque, New Mexico, USA; 2 University of New Mexico, Albuquerque, New Mexico, USA; 3 Atmospheric Research and Analysis Inc., Cary, North Carolina, USA; 4 Southern Company, Birmingham, Alabama, USA; 5 Georgia Institute of Technology, Atlanta, Georgia, USA; 6 University of Georgia, Athens, Georgia, USA; 7 SKS Consulting Services, Siler City, North Carolina, USA

**Keywords:** chemical mass balance, intratracheal instillation, *in vivo*, lung, particulate matter, PM_2.5_, projection to latent surfaces, source apportionment

## Abstract

**Background:**

Exposure to air pollution and, more specifically, particulate matter (PM) is associated with adverse health effects. However, the specific PM characteristics responsible for biological effects have not been defined.

**Objectives:**

In this project we examined the composition, sources, and relative toxicity of samples of PM with aerodynamic diameter ≥2.5 μm (PM_2.5_) collected from sites within the Southeastern Aerosol Research and Characterization (SEARCH) air monitoring network during two seasons. These sites represent four areas with differing sources of PM_2.5_, including local urban versus regional sources, urban areas with different contributions of transportation and industrial sources, and a site influenced by Gulf of Mexico weather patterns.

**Methods:**

We collected samples from each site during the winter and summer of 2004 for toxicity testing and for chemical analysis and chemical mass balance–based source apportionment. We also collected PM_2.5_ downwind of a series of prescribed forest burns. We assessed the toxicity of the samples by instillation into rat lungs and assessed general toxicity, acute cytotoxicity, and inflammation. Statistical dose–response modeling techniques were used to rank the relative toxicity and compare the seasonal differences at each site. Projection-to-latent-surfaces (PLS) techniques examined the relationships among sources, chemical composition, and toxicologic end points.

**Results and conclusions:**

Urban sites with high contributions from vehicles and industry were most toxic.

Concern over possible health effects of environmental particulate matter ≤2.5 μm (PM_2.5_) [U.S. Environmental Protection Agency (EPA) 2004] has stimulated numerous studies of its chemical/physical properties, the sources that contribute the most hazardous components, and biological mechanisms for the adverse effects. Although epidemiologic studies indicate that significant effects are often associated with PM_2.5_ exposure, the magnitude of the effect varies with location. *In vitro* studies have shown correlations between effects of PM and the contributing sources or composition (Alfaro-Moreno et al. 2002; Aust et al. 2002; Becker et al. 1996; Don Porto et al. 2001; Hatch et al. 1985; Huang et al. 2002; Imrich et al. 2000; Karlsson et al. 2005; Long et al. 2001; Maciejczyk and Chen 2005; Schins et al. 2004). Furthermore, *in vivo* studies have shown that effects of inhaled concentrated ambient particles (CAPS) vary with the daily CAPS composition (Clarke et al. 2000; Ghio and Huang 2004; Gurgueira et al. 2002; Saldiva et al. 2002; Schins et al. 2004), but such studies are limited to variation in composition and effect at single sites as a function of time. Other studies have examined the *in vitro* (Li et al. 2003) or *in vivo* (Dick et al. 2003) effects as functions of particle size. A well-known series of experiments compared the effects of materials collected from the Utah Valley during periods of operation or closure of a local steel mill (reviewed by Ghio 2004). Becker et al. (2005) examined the *in vitro* effects of PM from a single site as a function of season, and an epidemiologic study examined seasonal differences across 100 U.S. cities (Peng et al. 2005). However, few studies have directly compared the effects of ambient respirable PM from different locations *in vivo* (Gavett et al. 2003; Hatch et al. 1985). Such studies are critical to rational regulation of PM based on source/composition/toxicity relationships rather than size alone.

In the present study we used intratracheal instillation to compare toxicity of PM_2.5_ collected during summer or winter from four sites with different contributing sources. This technique, although a nonphysiologic method of administration, is useful for comparative studies in which the nature of collected samples precludes inhalation exposures (McDonald et al. 2004; Seagrave et al. 2002). We did not include *in vitro* analyses because we observed poor correlations with *in vivo* results for a series of engine exhaust samples (Seagrave et al. 2003).

The selected sites within the Southeastern Aerosol Research and Characterization (SEARCH) network represented a range of urban to rural areas with different contributing PM sources (Hansen et al. 2003). We collected PM_2.5_ during two seasons and performed source apportionment for these samples using the chemical mass balance (CMB) receptor model (Zheng et al. 2002). In addition to the SEARCH sites, we evaluated the toxicity of a sample collected downwind from a series of prescribed forest burns (smoke). Assessment of toxicity/site/composition relationships included relative toxicity rankings by site and projection-to-latent-surfaces (PLS) analysis (McDonald et al. 2004).

## Materials and Methods

### Site description

The selected sites represented a range of urban to rural areas in the southeastern United States with different contributing PM sources as previously described by Hansen et al. (2003). Briefly, the Birmingham, Alabama (BHM), site was an undeveloped building lot in an urban area, 3 km north of the downtown area (courthouse), within a few kilometers of heavy transportation and industry, including a coke production facility. The Jefferson Street, Atlanta, Georgia (JST), site was also an urban site located 4.2 km northwest of downtown Atlanta, amid parking lots, city streets, warehouses, and storage and within 250 m of a bus maintenance facility. The Pensacola, Florida (PNS), site was mixed urban and residential, near an elementary school, and 4.7 km from the Gulf of Mexico, whereas the Centreville, Alabama (CTR), site was rural and forested, proximal to the Talladega National Forest.

### Sample collection and processing

We collected ambient PM_2.5_ for toxicity testing on Teflon filters using high-volume samplers and extracted them by sonication, first with a 9:1 acetone:dichloromethane mixture and then with purified water. Both fractions were concentrated and combined to produce a 1:1 (vol/vol) acetone:aqueous mixture, thus reconstituting the atmospheric ratio of constituents. An extract control sample from unexposed filters was processed identically. Additional details of these methods are presented in Supplemental Material (available online at http://www.ehponline.org/docs/2006/9234/suppl.pdf).

### Chemical characterization of atmospheres and extracted samples

We collected parallel air samples to determine average atmospheric concentrations for each site/season and to estimate mass and species available for extraction on the filters for the toxicity testing. Briefly, we measured PM_2.5_ mass gravimetrically, trace elements by X-ray fluorescence (Hansen et al. 2003), sulfate and nitrate by ion chromatography, and ammonium by automated colorimetry. Organic carbon (OC) and elemental carbon (EC) were analyzed by thermal-optical reflectance at Desert Research Institute (Reno, Nevada) (Chow et al. 2001). Organic compounds were analyzed by gas chromatography/mass spectrometry (GC/MS) (Zheng et al. 2002). The Supplemental Material (available online at http://www.ehponline.org/docs/2006/9234/suppl.pdf) provides additional information on these methods.

The extracts generated for toxicity testing were also analyzed for selected constituents shown in previous studies to discriminate among sources.

### Source apportionment

We performed source apportionment based on the atmospheric chemistry using a CMB method previously described by Zheng et al. (2002). Briefly, chemical profiles of well-defined aerosol source emissions were defined by separate analyses. The chemical composition of the sample was then determined, and equations corresponding to linear combinations of the source profiles were solved using an effective variance-weighted least-squares analysis technique (Watson et al. 1984, 2001). The sources considered important for these sites included emissions from diesel and gasoline engines, wood combustion, paved road dust, meat cooking, vegetative detritus, natural gas combustion, and emissions from coke facilities (Zheng et al. 2006). Source profiles for wood combustion and paved road dust were modified as appropriate for the local composition of these sources (Zheng et al. 2002). Time-resolved and spatially resolved analyses of the sources of PM_2.5_ at the SEARCH sites are published separately (Zheng et al. 2006).

### *Measurement of* in vivo *toxicity*

#### Animals

Charles River Laboratories (Wilmington, MA) supplied the 8 ± 1-week-old male F344/Crl BR rats, which were quarantined for 3 weeks and confirmed free of common pathogens by serology. The rats, housed two per cage under a controlled light/dark cycle, temperature, and relative humidity conditions, had *ad libitum* access to food (Harlan Teklad Lab Blox; Harlan Teklad, Madison, WI) and water. The Institutional Animal Care and Use Committee approved all animal work, assuring humane use with regard for alleviation of suffering.

#### Reagents and supplies

All chemicals were obtained from Sigma Chemical Company (St. Louis, MO) unless otherwise specified. Acetone (optima grade) and dichloromethane (HPLC/GC-MS grade) were from Fisher Scientific (Fairlawn, NJ).

#### Sample preparation

We prepared PM_2.5_ suspensions and the extract control for instillation as previously described (Seagrave et al. 2002) as suspensions in vehicle (0.9% NaCl/ 1% acetone/0.01% Tween-80), with dilutions in the same vehicle. To confirm similar responsiveness among the different experimental series, we used National Institute of Standards and Technology (NIST; Gaithersburg, MD) standard reference material 2975 (forklift diesel soot) suspended in vehicle.

#### Intratracheal instillation

We instilled anesthetized rats (5% halothane in oxygen with nitrous oxide) with a sample or control material in 0.5 mL via a trans-oral cannula and returned them to their cages after recovery from anesthesia.

Each experimental series consisted of two samples at three doses (0.75, 1.5, and 3 mg/rat), the extract control, and the NIST diesel soot positive control, with five rats per dose. Because a significant fraction of each sample is soluble material, these doses would not be expected to cause overload phenomena (Oberdorster 1995). In addition, one series also included a group of uninstilled control rats.

#### Euthanasia and processing

We killed the rats with Euthasol (Virbac Labs, Ft. Worth, TX) 24 hr after instillation [the time of the maximal inflammatory and cytotoxic effects (Seagrave et al. 2002)] and recorded their body weights. Processing, lavage of the right lung lobes, and fixation were as previously described (Seagrave et al. 2002).

We evaluated total lavage cells using a hemacytometer and differential cell counts on Wright-Giemsa–stained cytocentrifuge preparations (Seagrave et al. 2002). We analyzed cell-free lavage fluid for lactate dehydrogenase (LDH) (Gay et al. 1968), total protein (Watanabe et al. 1986), and alkaline phosphatase (APase) using a Hitachi 911 (Roche Diagnostics, Basel, Switzerland) autoanalyzer.

A board-certified veterinary pathologist (A.P.G.) graded the lung histopathology. In accordance with guidelines of the Society of Toxicologic Pathologists (Crissman et al. 2004), we did not attempt a “blinded” evaluation. Furthermore, foreign matter was obvious in the lungs of treated animals. Responses were graded using a scale from 0 (normal) to 5 (extreme pathology: severe and widespread presence of a particular response/diagnosis). Each rat received scores summarizing responses in cytotoxic or inflammatory categories and a total score as previously described (Seagrave et al. 2002).

### Statistical analysis of toxicology data

We graphed the dose–response relationship for each sample. Responses to the extract control were similar for the series of experiments done for the winter and smoke samples, but these were slightly different from the responses to the extract control prepared in the experimental series to test the summer samples. Baseline values for the two series were therefore considered separately. As previously described, we fit an exponential function to the toxicity data and used the exponent of the equation (“potency factor”) to compare the toxicity of the samples (Seagrave et al. 2002). Using the entire dose–response curve provides substantially more statistical power to discriminate among samples than do individual dose-to-dose comparisons.

We evaluated differences among samples for each end point using *p*-values from pairwise *F*-tests, adjusted for multiple comparisons using the modified Bonferroni procedure of Hochberg (1988), with *p* = 0.05 as the criterion for statistical significance.

### PLS analysis

We used SIMCA (version 8; Umetrics Inc., Kinnelon, NJ) to perform a PLS analysis on the SEARCH site samples with the mass fractions of chemical classes as predictors and the toxicologic potency factors as responses. Because detailed organic speciation was not performed on the smoke sample, this sample was not included in the analysis. Table 1 shows the simplified organic composition classes used as predictors. OC was also considered as a separate predictor element, along with EC, ammonium, NO_3_^−^, SO_4_^2−^, arsenic, bromine, copper, manganese, lead, selenium, titanium dioxide, zinc, and a composite of metal oxides collectively referred to as major metal oxides (MMOs). Data were centered and scaled to unit variance before analysis. A second iteration of the analysis used the CMB-attributed sources as predictors. In the PLS analysis, the fraction of the total variation (*R*^2^) in the toxicologic responses and chemical constituent predictors was assessed for each component. A cross-validated cumulative prediction accuracy measure (*Q*^2^) was used to select the optimal number of components for the final models. Loading plots visually display the relationship between the predictors and responses as functions of the PLS components with the highest predictive capacity.

## Results

### Atmospheric chemistry

**Figure 1:**
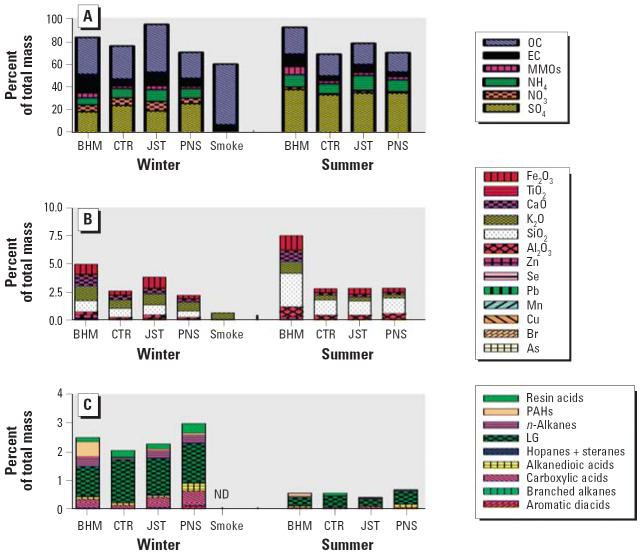
Atmospheric chemistry varies as a function of site and season. (*A*) Major classes of components (OC is not corrected for total OM). (*B*) Metals and MMOs. (*C*) Identified organic classes.

Analysis of the atmospheric chemistry showed both season- and site-related differences (Figure 1A,B). SO_4_^2−^, aluminum oxide (Al_2_O_3_), and silicon dioxide (SiO_2_) were higher at all sites during the summer, whereas OC, NO_3_^−^, and potassium oxide (K_2_O) were higher in winter. BHM-winter, BHM-summer, and JST-winter had the highest EC and ferric oxide (Fe_2_O_3_) levels. BHM-summer also had the highest levels of MMOs. The smoke sample contained predominantly OC; the only significant MMO in this sample was K_2_O.

Figure 1C shows the major classes of organic compounds as a percentage of the total mass. The organic mass (OM) fraction was higher in all sites in the winter. PNS-winter exhibited the highest fraction of many organic-compound classes, including alkane and aromatic diacids, branched alkanes, carboxylic acids, cholesterol, levoglucosan (LG), nonanal, and resin acids. However, BHM-winter had the highest levels of polycyclic aromatic hydrocarbons (PAHs), followed by BHM-summer, JST-winter, and PNS-winter. Cholesterol was highest at the PNS and JST sites, whereas hopanes and steranes were highest in JST-winter and BHM-winter, followed by PNS-winter and BHM-summer. The pattern for branched and straight alkanes was similar: highest in BHM-winter followed by the JST-winter and PNS-winter. CTR was noteworthy in having the lowest levels of *n*- and branched alkanes, hopanes and steranes, alkane and aromatic diacids, and PAHs in both seasons, but in the summer it had the highest resin acids.

### Source apportionment

**Figure 2:**
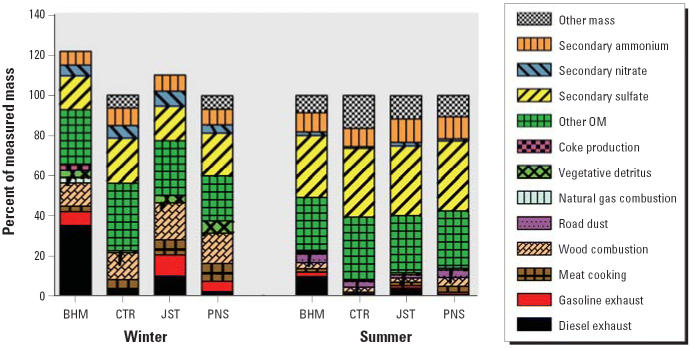
Reconstructed PM_2.5_ mass of winter and summer samples. The major sources are described for the four SEARCH sites for two seasons.

Figure 2 shows results of the CMB analysis for the SEARCH sites. As expected, wood smoke and secondary NO_3_^−^ contributed more mass to the winter samples. In contrast, summer samples contained more secondary SO_4_^2−^. Diesel exhaust was a minor component of the CTR and PNS samples (both seasons) but contributed substantially to the mass in the urban/industrial sites, especially BHM-winter. Gasoline emissions were also quite high in BHM-winter and JST-winter. Meat cooking contributed more to the mass in the winter, except for BHM, whereas road dust was significant only in the summer. Unidentified OM (other OM), which includes secondary organic aerosol, was substantial in all sites in both seasons but was generally greater in summer.

### Sample chemistry

**Figure 3:**
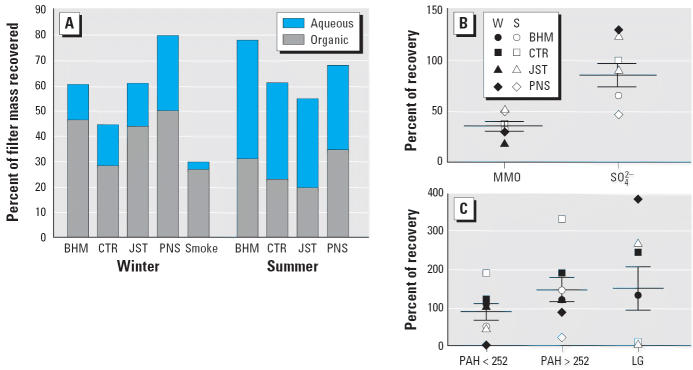
Sample chemistry: recovery based on total mass of the analytes measured in extracts for toxicity testing relative to parallel samples collected for atmospheric chemistry. (*A*) Mass recovered in the organic solvent and aqueous extractions shown as the percentage of the total mass on the filters. (*B*) Percent recovery (mean and SE) of the two largest classes of inorganic materials (MMOs and SO_4_^2−^) relative to parallel atmospheric samples. (*C*) Percent recovery (mean and SE) of PAHs and LG relative to parallel atmospheric samples. Abbreviations: S, summer; W, winter.

Mass recovered in the extracts for toxicity testing averaged 60% of the total mass estimated from the filter loading of parallel filters collected for the chemical analyses with a somewhat lower recovery from the smoke sample. The organic solvent extracted a larger fraction of the collected mass for the winter and smoke samples, whereas the aqueous extract contained more of the mass from the summer samples (Figure 3A).

NO_3_^−^ was not detected in any of the winter extracts, possibly due to losses during storage between sample extraction and analysis (Schaap et al. 2006). Examples of the recovered mass relative to the predicted mass for selected inorganic and organic analytes are presented in Figure 3B and C. Not surprisingly, the largest discrepancies were observed in analytes with the lowest starting masses (e.g., LG in the summer samples). Recovery of MMOs was around 50% for all samples. Recoveries > 100% were occasionally observed, possibly due to methodologic differences. However, the range of recoveries was rarely > 2-fold, whereas the range in actual masses among the different samples was much greater, and thus the rank order of the samples was usually preserved through the extraction process.

### Toxicity

**Figure 4:**
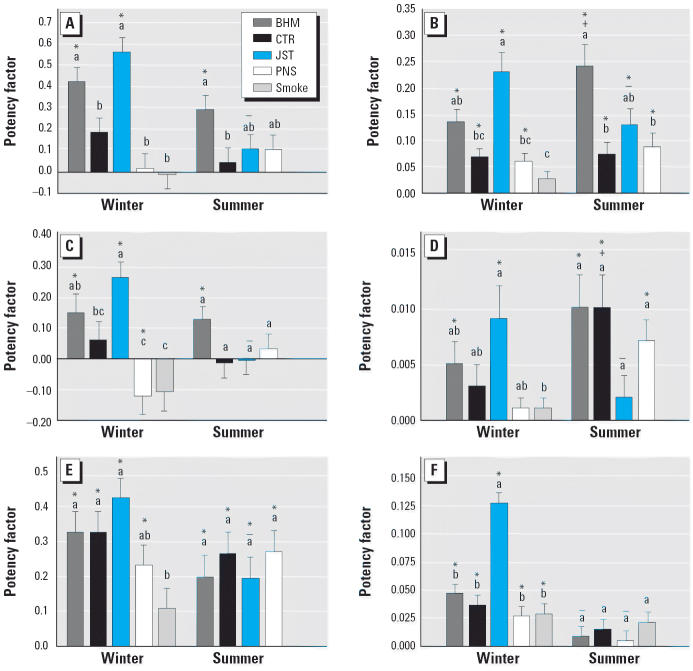
Inflammatory potency varies with site and season. (*A*) Total cells. (*B*) Polymorphonuclear leukocytes. (*C*) Macrophages. (*D*) Lymphocytes. (*E*) Histopathologic inflammation. (*F*) Lung weight:body weight ratio. Bars represent potencies for the inflammatory end points (mean and SE). Letters above the bars indicate samples within a season that are not significantly different from each other; “+” and “–” indicate potency significantly greater than and less than, respectively, the potency of the sample from the same site in the winter. *Potency significantly different from 0.

**Figure 5:**
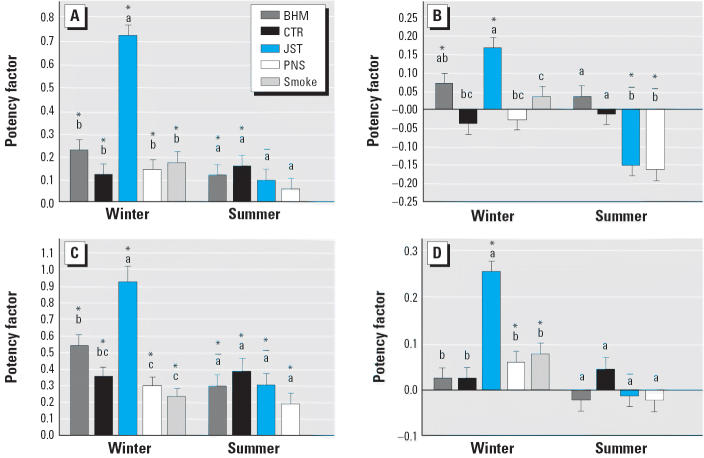
Cytotoxicity potency varies with site and season. Bars represent potencies for the inflammatory end points (mean and SE). (*A*) LDH. (*B*) APase. (*C*) Protein. (*D*) Histopathologic cytotoxicity. Letters above the bars indicate samples within a season that are not significantly different from each other; “–” indicates potency significantly less than the potency of the sample from the same site in the winter. *Potency significantly different from 0.

Figures 4 and 5 show the potency factors for the inflammatory (including lung weight:body weight ratio) and cytotoxic parameters, respectively. Among the samples collected in the winter, JST-winter caused significantly more toxicity (LDH, APase, total protein, and histopathologic cytotoxicity and increases in lung weight) than the other winter samples. BHM-winter was the second most potent for these indicators except histopathology and was significantly more potent than PNS-winter or CTR-winter for increases in APase and total protein. JST-winter also most potently induced inflammation. It was significantly more potent than CTR-winter, PNS-winter, and smoke for total cells, neutrophils, macrophages, and lung weight:body weight ratio and significantly greater than smoke for lymphocytes and histopathologic indication of inflammation. Although JST-winter was not significantly different from BHM-winter, BHM-winter was significantly more potent than the other samples for total cells and significantly more potent than smoke for neutrophils, macrophages, and histopathologic inflammation. PNS-winter caused a statistically significantly negative potency for macrophages. The smoke sample had a similar effect that did not reach statistical significance.

There were smaller differences among the summer samples. Among the toxicity indicators, only APase demonstrated significant differences among the samples: JST-summer and PNS-summer suppressed this enzyme activity. All summer samples significantly increased neutrophils, with BHM-summer being significantly more potent than CTR-summer and PNS-summer. BHM-summer also significantly increased macrophages, although the response was not significantly different from the other summer samples.

Interestingly, JST-summer was significantly less potent than JST-winter for all end points. The potency of BHM-summer was also less than BHM-winter for most end points, but only the effect on protein reached statistical significance. In contrast, CTR-summer was more potent than CTR-winter for lymphocytes. Similarly, BHM- and PNS-summer increased lymphocytes more than the corresponding winter samples, although the differences were not statistically significant. The only significant difference between BHM-winter and BHM-summer was the greater suppression of APase by the summer sample.

### PLS analysis

**Figure 6:**
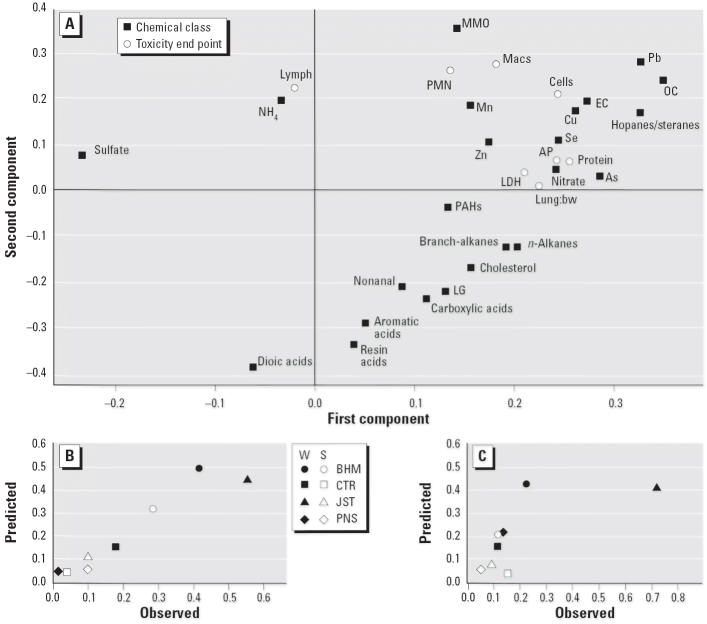
PLS analysis based on chemical classes. (*A*) Loading plot showing relationships among predictors (chemical class) and responses (toxicity end points) based on a two-component model. Observed versus predicted responses for (*B*) total cells and (*C*) lavage LDH. Abbreviations: S, summer; W, winter.

The PLS analysis using the chemical predictors identified two major components, which explained 64% of the total variation in the responses and 77% of the total variation in the predictors. Although *R*^2^ is reasonably high for several of the responses (0.7 for APase, 0.9 for cells, 0.7 for protein), *Q*^2^ is low (0.35, 0.3, and 0.35, respectively). Figure 6A shows a loading plot. The most important predictors were OC, Pb, and hopanes/steranes, with NO_3_^−^ and As strongly influencing the first component, and MMOs influencing the second component. The first component more strongly affected the cyto-toxic responses, whereas the second component more strongly affected the inflammatory responses (except lymphocytes). Predicted versus observed results for total cells and LDH, respectively, are presented in Figure 6B and C.

**Figure 7:**
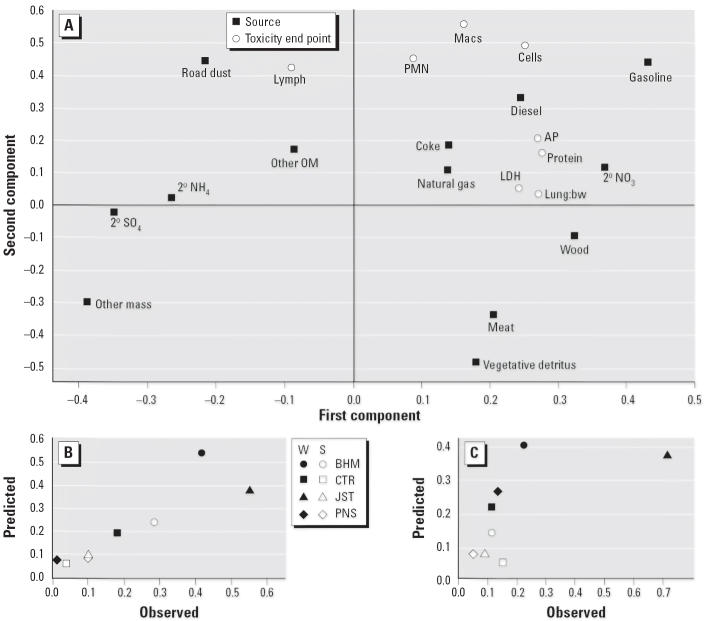
PLS analysis based on source apportionment. (*A*) Loading plot showing relationships between predictors (sources) and responses (toxicity end points) based on a two-component model. Observed versus predicted responses for (*B*) total cells and (*C*) lavage LDH. Abbreviations: S, summer; W, winter.

A second analysis using the source apportionment results again indicated that two components were sufficient to explain 77% of the overall variation in the predictors and 52% of the overall variation in the responses. *R*^2^ and *Q*^2^ for the response predictions were similar to the analysis using all chemical variables. The loading plot (Figure 7A) shows that gasoline emissions were the most important predictor (both components), whereas diesel more strongly influenced the second component and secondary NO_3_^−^ influenced primarily the first component. The loading plot again suggests a greater influence of the first component on cytotoxic responses and of the second component on inflammatory responses. Predicted versus observed results for total cells and LDH are shown in Figure 7B and C.

## Discussion

This study showed that the biological effects of intratracheal instillation of equivalent masses of PM_2.5_ differ as a function of site and season, thus implicating specific constituents and/or sources in its effects. Although this is intuitively reasonable and is supported by other experimental evidence, current air quality regulations are based only on mass in specific size fractions. Identification of the most potent constituents should lead to more targeted regulation to protect populations at risk.

Intratracheal instillation of collected and extracted samples has limitations, including the high doses usually used. Furthermore, the non-physiologic route of administration results in deposition of all particle sizes with the same spatial distribution, which may be nonuniform and different from that achieved by inhalation. However, this method is very useful for preliminary screening studies for direct comparisons of multiple materials (Costa et al. 2006; Driscoll et al. 2000; Seagrave and McDonald 2004; Warheit et al. 2005). Another limitation is that recovery of the mass from filters used to collect the PM_2.5_ is rarely 100% efficient. Therefore, if the extraction is selective, leaving behind more or less toxic constituents, the toxicity results may underestimate or overestimate (respectively) the toxicity of the original material. It is therefore important to optimize the extraction and, where possible, compare the composition of the extracted material with the original filter samples. Extraction of these ambient samples included a secondary aqueous extraction not included in the previous studies of engine emission samples (Seagrave et al. 2002, 2005a). The aqueous extract contained substantial additional mass, particularly for the summer samples, most likely due to increased NH_4_SO_4_ in these samples. However, interpretation must be tempered by the fact that 100% recovery was not achieved.

Wood smoke can be a significant contributor to ambient PM_2.5_ mass, especially in the winter. Previous studies have indicated potential health effects of relatively high concentrations of smoke (Barrett et al. 2006; Boman et al. 2003; Burchiel et al. 2005; Park et al. 2004; Seagrave et al. 2005b; Tesfaigzi et al. 2002, 2005; Zelikoff et al. 2002). However, we observed little toxicity from the smoke sample, which consisted of relatively fresh smoke from prescribed forest burns (primarily smoke from forest understory: live or dead branches, stumps, leaves, pine needles, shrubs, and grass). In contrast, the wood smoke in the SEARCH site samples was most likely from aged fireplace and woodstove emissions. Given the lack of effect of the smoke sample, in combination with the fact that neither the chemicals associated with wood smoke nor the wood smoke source from the CMB apportionment correlated with the toxicity in the PLS analyses, it seems unlikely that wood smoke PM_2.5_ contributed significantly to the toxicologic responses.

The winter samples from the two more-urban/industrial sites produced the greatest responses, with JST-winter being significantly more potent than BHM-winter for several of the cytotoxicity responses. BHM-summer and BHM-winter were similar in potency, but JST-summer was significantly less potent than JST-winter for most end points. The ambient composition for the sites from which the most potent samples were collected includes higher levels of EC, *n*-alkanes, hopanes and steranes, and NO_3_^−^. However, NO_3_^−^ was not detectable in the winter extracts, so it is unlikely that NO_3_^−^ could have contributed to the toxicity. PAHs were also higher in both BHM samples, but JST-winter and PNS-winter were similar for this class of chemicals.

Source apportionment suggested that the three most potent samples include more PM_2.5_ from diesel and gasoline exhaust. The impact of these emissions is supported by the PLS analysis.

A limitation to these PLS analyses is the poor prediction capacity of *Q*^2^, which reflects the sensitivity of the analysis to inclusion of individual samples and the large number of chemical constituent predictor variables relative to the small number of samples (eight). In addition, poor prediction capacity could also indicate that the most toxic constituents were not measured or that variation in extraction efficiency interfered with the composition/ toxicity correlation. Although PLS analysis using the attributed sources introduces an additional level of uncertainty, the results of this analysis generally support the analysis using the primary chemical composition.

In summary, this study supports the concept that PM_2.5_ composition affects its toxicity. Specifically, the most toxic samples were from the sites during seasons with the largest contributions of diesel and gasoline emissions, whereas wood burning was only weakly correlated with toxicity end points. The PLS analysis also indicated that SO_4_^2−^, secondary organic aerosols, meat cooking, and vegetative detritus were not correlated with the biological responses.

## Figures and Tables

**Table 1:** Chemical classes and key sources.

Compound	Source
Organic	
*n*-Alkanes	Vegetative detritus, vehicles (diesel)
Branched alkanes	Vegetative detritus, motor vehicles
Alkane dioic acids	Secondary organic aerosol
Aromatic dioic acids	Secondary organic aerosol
Benz(de)anthracene-7-one	Coke, other combustion
Carboxylic acids	Combustion sources, vegetative detritus, microbes
Cholesterol	Meat cooking
Hopanes and steranes	Vehicle emissions, lube oil
LG	Wood combustion
Nonanal	Meat cooking
PAHs	Combustion (wood, coke, motor vehicles)
Resin acids	Wood combustion
OC	Combustion (wood, meat, motor vehicles)
Inorganic	
Ammonium	Agriculture/livestock and gasoline exhaust
EC	Diesel, other combustion
MMOs and other metals	Resuspended (road) dust
Manganese	Motor vehicles and road dust
NO_3_^−^	Combustion (wood, meat, motor vehicles, coal)
Lead	Motor vehicles and road dust
SO_4_^2−^	Combustion (coal, motor vehicles, others)
Zinc	Motor vehicles and road dust
