# Supplementary material for: Lung Toxicity of Ambient Particulate Matter from Southeastern U.S. Sites with Different Contributing Sources: Relationships between Composition and Effects
Source: Environ Health Perspect. 2006 Jun 12;114(9):1387–93. doi: 10.1289/ehp.9234 (PMC1570075; doi:10.1289/ehp.9234)
Supplement: Supplemental Figures and Tables [file ehp0114-001387s1.pdf]

Sample Collection and Processing

Ambient $PM_{2.5}$ was collected for toxicity testing on 8 x 10 in Zefluor filters (Pall Corp, East Hills, NY) using high volume samplers (Tisch, Inc., Village of Cleves, OH) equipped with 2.5 μm size-selective inlets (Dp_{50} = 2.5 μm, sharpness = 1.33). Samples were collected for 24-hr periods (midnight to midnight) every third day at a flow rate of 1.13 m^3/min during November 2003 – January 2004 (winter) and July – September 2004 (summer). In addition to the SEARCH sites, a sample collected downwind from a series of prescribed forest burns (Smoke) during February – April 2004 was collected for toxicity testing. The filters were stored in sealed polyethylene bags at -15°C until shipment (on previously frozen "Blue Ice" packs) to Lovelace Respiratory Research Institute, where they were stored at -20°C prior to extraction.

Extraction was accomplished by first immersing the filters in a mixture of acetone and dichloromethane (DCM) (9:1) in a glass dish and moving the tip of 3 cm wide probe sonicator in a regular pattern to release the particles. Although this procedure appeared to release the majority of the insoluble particles into the suspension, preliminary studies showed that organic extraction alone did not efficiently remove inorganic sulfates and nitrates which account for a large portion of the mass of these samples, so aqueous-soluble constituents were then extracted into MilliQ purified (> 18 MΩ) water by bulk sonication in a sonic bath. The acetone/DCM suspension was concentrated by evaporation under nitrogen and the aqueous fraction was concentrated by lyophilization. Masses of the material recovered in each fraction were determined from the residual weights of known volumes of the suspensions after evaporation of the solvent at room temperature.

The two fractions were concentrated to equal volumes and the fractions were combined to produce a 1:1 acetone:aqueous mixture at a concentration of 120 mg/ml total sample mass. Unexposed filters were processed similarly to provide an extract control sample.

Chemical Characterization of Atmospheres and Extracted Samples

Parallel air samples were collected to (1) determine atmospheric concentrations during each field campaign and (2) estimate mass and species loadings on the Zefluor filters used for the toxicity testing (i.e., mass/species available for extraction). Low-volume, 24-hr samples for $PM_{2.5}$ mass and trace elements were collected on 2 micron pore size, 47 mm diameter ringed Teflon filters (Whatman, Middlesex, UK) using a Federal Reference Method sampler (Model 2025, Rupprecht & Patashnick, East Greenbush, NY) equipped with a WINS impactor (Dp_{50} = 2.5 μm, sharpness = 1.16). Collected mass and trace elements were determined gravimetrically and by x-ray fluorescence (XRF), respectively. Samples for ionic (sulfate, nitrate, ammonium) and organic and elemental carbon (OC/EC) analysis were collected on 47 mm Teflon and 37 mm pre-baked quartz filters (provided by Desert Research Institute, DRI, Reno, NV), respectively, using a multi-channel, sequential $PM_{2.5}$ sampler (ARA, Inc., Plano, TX), also equipped with a WINS impactor. Teflon filters were extracted by shaking in 25 mL of deionized water for 45 min and then analyzed for sulfate and nitrate by ion chromatography, ammonium by automated colorimetry, and metals by XRF (Hansen et al. 2003). Quartz filters were analyzed for OC and EC by DRI using the thermal-optical reflectance method (Chow et al. 2001). Data for all samples were combined to estimate content in the extracts for toxicity testing.

High-volume $PM_{2.5}$ samples for detailed organic compound analysis were collected on 20.3 x 25.4 cm pre-baked quartz filters using a Tisch Environmental Model TE-6001 sampler. Following collection, a circular punch was removed from each filter (10 - 24 filters per site/season) and the punches were composited for analysis. Filter composites were extracted three times in benzene/2-propanol, derivatized with diazomethane, then analyzed for >100 organic compounds via gas chromatography/mass spectrometry and quantified using authentic

standards from Wisconsin State Laboratory of Hygiene (Madison, WI) as described elsewhere (Zheng et al. 2002).

The organic and aqueous suspensions generated for toxicity testing were also chemically analyzed for a subset of analytes, focusing on chemicals used in previous studies to illustrate differences among motor vehicle and other types of emissions.

Reference List

1. Hansen DA, Edgerton ES, Hartsell BE, Jansen JJ, Kadasamy N, Hidy GM et al. 2003. The southeastern aerosol research and characterization study: Part 1-Overview. J Air Waste Manag Assoc 53:1460-1471.

2. Chow JC, Watson JG, Crow D, Lowenthal DH, Merrifield T. 2001. Comparison of IMPROVE and NIOSH carbon measurements. Aerosol Sci Technol 34:23-34.

3. Zheng M, Cass GR, Schauer JJ, Edgerton ES. 2002. Source apportionment of PM2.5 in the Southeastern United States using solvent-extractable organic compounds as tracers. Environ Sci Technol 36:2361-2371.
